# First Report of a Deletion Encompassing an Entire Exon in the Homogentisate 1,2-Dioxygenase Gene Causing Alkaptonuria

**DOI:** 10.1371/journal.pone.0106948

**Published:** 2014-09-18

**Authors:** Mohammad Zouheir Habbal, Tarek Bou-Assi, Jun Zhu, Renius Owen, Farid F. Chehab

**Affiliations:** 1 Department of Pathology and Laboratory Medicine, American University of Beirut, Beirut, Lebanon; 2 Department of Laboratory Medicine, University of California San Francisco, San Francisco, California, United States of America; 3 Quest Diagnostics, Nichols Institute, San Juan Capistrano, California, United States of America; Cancer Research Centre of Lyon, France

## Abstract

Alkaptonuria is often diagnosed clinically with episodes of dark urine, biochemically by the accumulation of peripheral homogentisic acid and molecularly by the presence of mutations in the homogentisate 1,2-dioxygenase gene (HGD). Alkaptonuria is invariably associated with HGD mutations, which consist of single nucleotide variants and small insertions/deletions. Surprisingly, the presence of deletions beyond a few nucleotides among over 150 reported deleterious mutations has not been described, raising the suspicion that this gene might be protected against the detrimental mechanisms of gene rearrangements. The quest for an HGD mutation in a proband with AKU revealed with a SNP array five large regions of homozygosity (5–16 Mb), one of which includes the HGD gene. A homozygous deletion of 649 bp deletion that encompasses the 72 nucleotides of exon 2 and surrounding DNA sequences in flanking introns of the HGD gene was unveiled in a proband with AKU. The nature of this deletion suggests that this in-frame deletion could generate a protein without exon 2. Thus, we modeled the tertiary structure of the mutant protein structure to determine the effect of exon 2 deletion. While the two β-pleated sheets encoded by exon 2 were missing in the mutant structure, other β-pleated sheets are largely unaffected by the deletion. However, nine novel α-helical coils substituted the eight coils present in the native HGD crystal structure. Thus, this deletion results in a deleterious enzyme, which is consistent with the proband’s phenotype. Screening for mutations in the HGD gene, particularly in the Middle East, ought to include this exon 2 deletion in order to determine its frequency and uncover its origin.

## Introduction

Tyrosine, a common residue of receptor tyrosine kinases, is a critical phosphorylation target in signal transduction pathways. It is also a precursor for the biosynthesis of the neurotransmitters dopamine and epinephrine. The regulation of neurotransmitters is in part regulated by the breakdown of tyrosine into its simple constituents acetic and fumaric acids. A key step in the catabolism of tyrosine is the decyclization of homogentisic acid to 4-maleylacetoacetate. This reaction is catalyzed by homogentisic acid dioxygenase (EC.1.13.11.5), a hepatic enzyme that when deficient results in the urinary excretion of large accumulated amounts of homogentisic acid and its deposition in various tissues. Alkaptonuria (AKU; OMIM #203500), a rare autosomal recessive disorder resulting from mutations in the homogentisate 1,2-dioxygenase (HGD) gene, has a wide spectrum of clinical manifestations. It is mainly characterized by the appearance of dark brown urine, which is derived from the oxidation of homogentisic acid into benzoquinones. Deposition of benzoquinones in connective tissues results in the darkening of the sclera, ear cartilages, causing arthritis. Other manifestations include cardiac involvement, coronary artery calcification and kidney stones [1]. Currently, there is no effective treatment for this condition. However, studies have shown that nitisinone could significantly reduce urinary homogentisic acid [2] by inhibiting 4-hydroxyphenylpyruvate dioxidase, the enzyme that produces homogentisic acid.

The HGD gene, located on chromosome 3 at hg19 coordinates 120,347,020–120,401,418, is expressed in the kidneys, liver, prostate, small intestine, colon and expresses a 1,715 nucleotide transcript that encodes 445 amino acids [3]. Mutations in the human HGD gene have been identified among patients in more than 40 countries throughout the world [4], [5]. The majority of these mutations, which consist mostly of missense mutations, nonsense mutations, splice sites and small insertions/deletions tend to aggregate particularly in exons 6, 8, 10 and 13 [6]. In this communication, we describe a novel 649 bp deletion that encompasses exon 2 of the HGD gene in a Lebanese individual, reported to have alkaptonuria and Pompe’s disease. While earlier attempts to uncover the HGD mutation have failed [7], we embarked on the characterization of this mutation with the hope to uncover a molecular lesion that could be used for the screening of similar affected patients in this area.

## Methods

### Human subjects research

An Institutional Review Board of the American University of Beirut approved the conduct of these studies and informed written consent was obtained from the parents, who also signed a form on behalf of their minor child.

### SNP microarray testing

DNA was extracted according to standard procedures using a proteinase K based protocol followed by ethanol precipitation. One microgram of DNA from the affected child was tested using the Affymetrix CytoScan HD SNP microarray to interrogate 750,000 single nucleotide polymorphisms (SNP) across the human genome. DNA amplification, hybridization, and washing of the microarray followed by high-density scanning were performed according to the manufacturer’s recommendations. The resulting data were analyzed with the Affymetrix Chromosome Analysis Suite (CHAS) software. The resulting data have been deposited in the ArrayExpress repository as accession number E-MTAB-2792.

### PCR, DNA sequencing and sequence analysis

Polymerase chain reaction (PCR) amplification of the region surrounding exon 2 of the HGD gene was carried out with the forward (5′ TCAGGGGCTCTCCATGACTT 3′) and reverse (5′ CTAACCAGCACTAGTATGAACATC 3′) primers. PCR cycling conditions on an Applied Biosystems 7100 thermocycler, consisted of 30 sec. each at 95°C, 55°C and 72°C for 35 cycles with 75% ramping times for the annealing and extension steps. PCR products were then separated on a 1.5% agarose gel and stained with ethidium bromide. For DNA sequencing, the PCR products were purified by spin-column chromatography and used as templates for Sanger dideoxy DNA sequencing. The sequencing products were fractionated and detected on an Applied Biosystems 3100 capillary electrophoresis DNA sequencing instrument. DNA sequence analysis was subsequently performed with the CodonCode Aligner software.

### 3-D Modeling of the HGD Mutant Protein

Structure prediction and three-dimensional modeling of the mutant HGD protein was determined by the full-chain protein structure prediction server Robetta [8], which parses protein chains into putative domains with the Ginzu protocol, and models those domains by homology to other HGD proteins. The crystal structure of the normal human HGD protein was obtained from the Protein Data Bank (PDB; 1EY2). Both normal and mutant protein structures were viewed with the MacPymol version1.07 (www.pymol.org) software.

## Results

### SNP microarray

**Figure 1:**
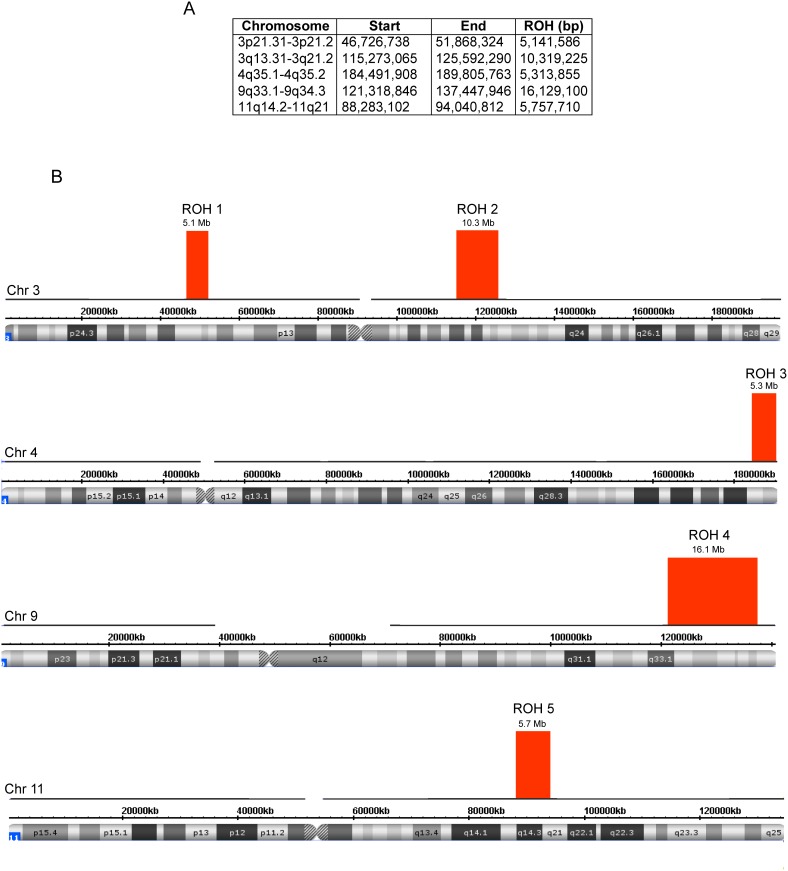
Regions of homozygosity (ROH) derived from the proband in the SNP array. (**A**) The cytogenetic location, genomic coordinates (hg19) and sizes of each ROH. (**B**) Location and size (in Mb) of each ROH on chromosomes 3, 4, 9 and 11.

The clinical findings of this patient were previously described, however attempts to uncover a deleterious mutation in the HGD gene were unsuccessful [7]. The SNP microarray revealed three regions of homozygosity (ROH) on chromosomes 3, 4 and 11 that span 5.1 Mb, 5.3 Mb and 5.7 Mb, respectively. In addition two other regions on chromosome 3 and 9 encompassed 10.3 Mb and 16.1 Mb, respectively (Fig. 1). There were no heterozygous calls in any of those regions. Altogether, the ROH totaled approximately 42.5 Mb encompassing 1.475% of the human genome, thus placing the parents putatively as third cousins (consanguinity factor F = 1/64). The 5.1 Mb ROH on chromosome 3 included the HGD gene, suggesting the presence of a homozygous mutation in this gene.

### PCR, DNA sequencing and analysis of the breakpoint regions

**Figure 2:**
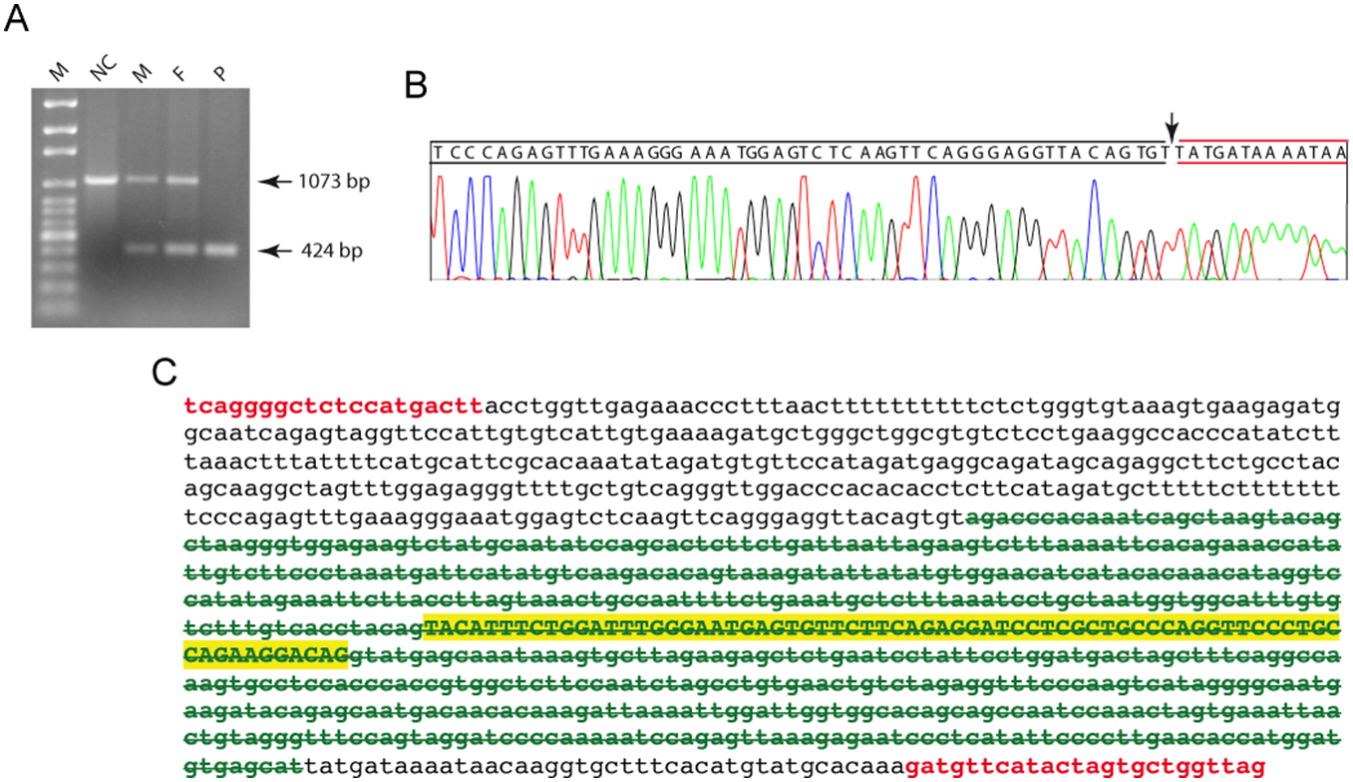
Identification of the 649 bp deletion in the proband. (**A**) PCR genotyping of the 649 bp deletion in a normal control (NC), mother (M), father (F) and proband (P). Size markers (M) consist of a 500 bp ladder. The sizes of the amplicons representing the normal (1073 bp) and mutant (424 bp) alleles are indicated next to the gel. (**B**) Sanger DNA sequencing electropherogram of the mutant amplicon, showing the deletion breakpoint (indicated by the arrow). (**C**) Representative DNA sequence of the 1073 bp amplicon extending from the forward and reverse primer (shown in red color). The strikethrough bases indicate the deleted DNA sequences and those of exon 2 are highlighted.

Targeted PCR of HGD exons in the proband revealed a lack of amplification of exon 2 with PCR primers adjacent to the splice sites. However, a shorter than normal PCR product was detected with PCR primers distant from the splice sites, revealing a deletion encompassing exon 2 (Fig. 2A). Follow-up Sanger based DNA sequencing of the mutant PCR product (Fig. 2B) revealed at chromosome 3 coordinates 120,394,334-120,394,982 (hg19), the presence of a 649 bp homozygous contiguous deletion (g.11347-11995del) that includes 272 bp of intron 1, the entire 72 bp of exon 2 and 305 bp into intron 3 (Figure 2C). Thus, the effects of this mutation include deletion of the acceptor site in intron 1, the splice donor site of intron 2 and at the protein level would be denoted by p.Tyr6Gln29del. This variant has been submitted to the mutation repository ClinVar (accession SCV000172498) and annotated according to the Human Genome Variation Society (HGVS) as NG_011957.1:g.11347_11995del and NM_000187.3:c.16-272_87+305del.

### Effect of the exon 2 deletion on the tertiary HGD structure

**Figure 3:**
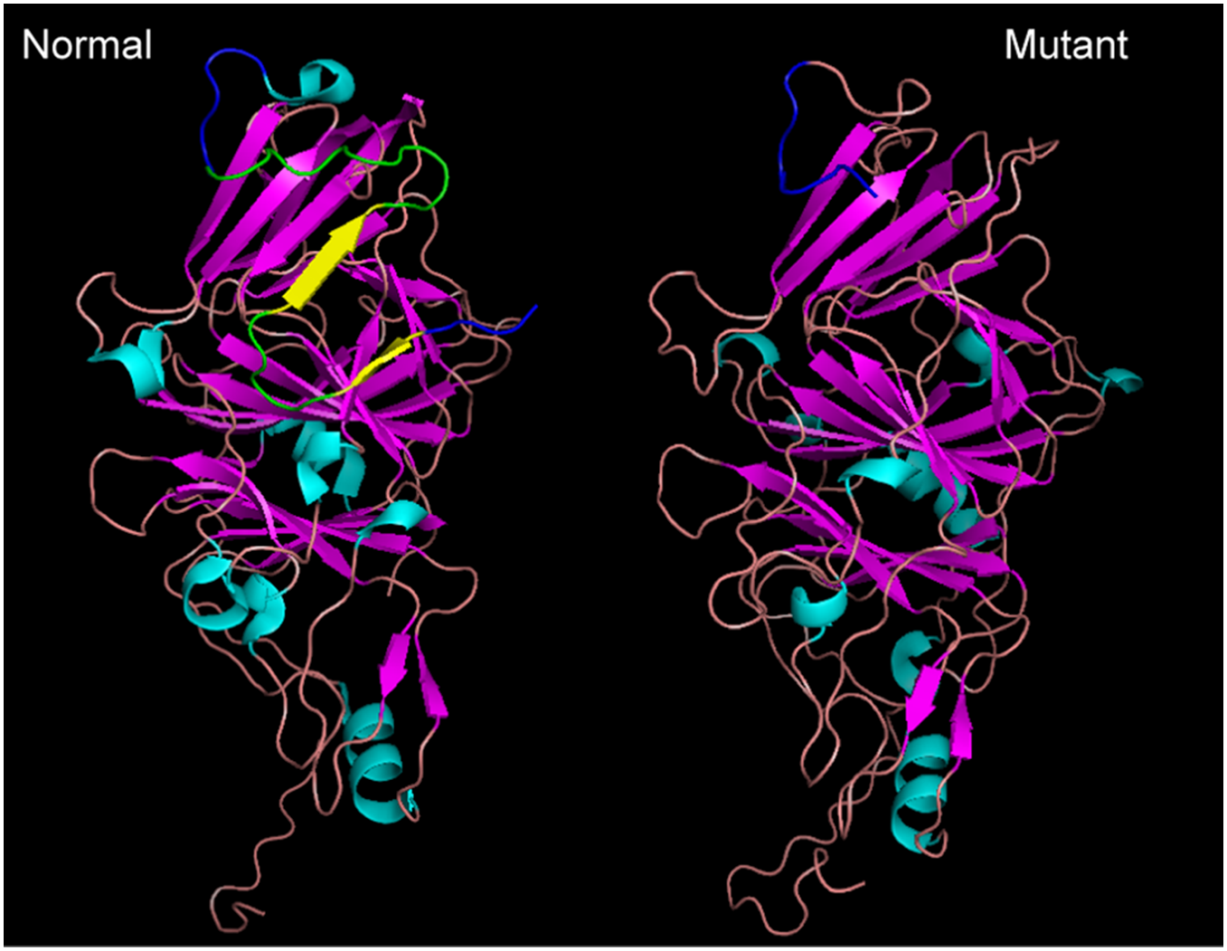
Tertiary structure modeling of the mutant HGD protein. (**A**) Crystal structure of the normal and (**B**) tertiary structure model of the mutant HGD protein α-helical coils, β-pleated sheets and interconnecting loops are colored in turquoise, magenta and pink, respectively. In (A), exon 2 is represented by the two yellow anti-parallel β-pleated sheets and green interconnecting loops. The adjoining four amino acids from exons 1 and 3 are shown in blue. Note in (B), the absence of exon 2 β-pleated sheets and loops and the presence of a novel loop (shown in blue) that represents 4 amino acids from each of exons 1 and 3. Despite the similarities of the β-sheets between the two structures, most of the amino acids in the α-helical coils are different from the native structure. This figure does not contain any copyrighted image.

Crystal structure of the native HGD protein revealed 8 α-helical coils, 28 β-pleated sheets and 31 interconnecting loops. Exon 2 is represented in the HGD wild-type protein by two anti-parallel β-pleated sheets encoded by the amino acids YISG and CSSE that are connected by the loop with the amino acids FGNE. The first 12 amino acids of the adjacent loop, DPRCPGSLPEGQNNPQV (Fig. 3A) are also encoded by exon 2. Prediction analysis of the secondary and tertiary structures of the mutant HGD protein resulted, as expected, in the deletion of the two β-sheets and two loops encoded by exon 2 (Fig. 3B). In addition, 9 new α-helical coils were predicted in the mutant protein and were different than those of the native protein (Table 1). While 4 of the 28 β-pleated sheets were missing in the mutant HGD and a new one predicted, other β-pleated sheets were either identical or highly similar to the native protein. Thus, the deletion is expected to significantly disrupt the tertiary structure of the native HGD enzyme and to compromise its catalytic activity.

**Table 1:** Amino acid sequences of α-helical coils and β-pleated sheets in the normal and mutant HGD proteins are listed from their amino to carboxy termini.

	Normal HGD	Mutant HGD
**α-helical coils**	CPYNL	WDE
	PRSTNK	ASQ
	LGPIG	FVSG
	NPR	IKS
	KNF	PRD
	DPSI	PSIF
	DADCFEKASKV	ADCFEKASK
	TKWGLKAS	KWGLKASR
	absent	YHKC
**β-pleated sheets**	YIS	deleted
	CSSE	deleted
	YAEQLS	YAEQLS
	RSWLYR	RSWLYR
	FES	absent
	LRW	LRW
	LHTLCGAGD	LHTLCGA
	NGLAIHIFLC	GLAIHIFLC
	RCFYN	RCFYN
	GDFLIVPQK	GDFLIVPQK
	LLIYT	LLIYT
	GKMLV	KMLV
	EICVI	EICVIQ
	FSID	FSID
	TRGYILEVY	TRGYILEVY
	FLIP	absent
	QVPGGYTVINKY	YTVINKY
	KLFAAKQDV	KLFAAKQ
	VVAWHG	VAWHG
	YKYN	YKYN
	TVLTAK	VLTAK
	AIADFVIF	IADFVIF
	RWG	RWG
	SEFMSEGLIR	SEFMGLIR
	GGGSLH	GGGSLH
	ERI	ERI
		MAFMFES

Note that all the coils in the predicted protein are different whereas only some β-pleated sheets are missing. The two pleated sheets spanned by exon 2 are denoted as deleted from the mutant protein.

## Discussion

We hereby report the characterization of a novel mutation causing AKU in a Lebanese child. While initial attempts to uncover an HGD mutation resulted in three non-deleterious polymorphisms only, it is likely that this deletion escaped detection by the initial PCR strategy [7]. In this study, the unveiling of the three regions of homozygosity are consistent with a third cousin consanguineous mating, suggesting the presence of a homozygous mutation in this autosomal recessive disorder. The uncovering of the 649 bp deletion encompassing all the 72 bp of exon 2 is the first example of a deletion that spans an entire exon in the HGD gene, in which over 150 point mutations and small insertions/deletions were previously identified [6], [9].

Because the deletion encompasses the surrounding splice donor and acceptor sites and 272 bp of intron and 305 bp of intron 3, it would result in an in-frame deletion that links exons 1 and 3 without exon 2. Thus, it is conceivable that a mutant mRNA without exon 2 could be transcribed and spliced, resulting in a protein with full or limited functionality. If such a protein were to be synthesized, its enzymatic activity is likely to be compromised, as evidenced by the accumulation of homogentisic acid in the proband [7]. Modeling of the mutant HGD protein with the 649 bp deletion resulted in an alternate structure that lacked from the native protein [10] two β-pleated sheets and an interconnecting loop, both encoded by the deleted exon 2. Furthermore, substitution of the eight α-helical coils with nine novel ones was characteristic of the predicted mutant HGD structure. Interestingly, most β-pleated sheets and interconnecting loops remained similar to the native protein, suggesting that the major impact of the exon 2 deletion is on the α-helical coils. In addition, the catalytic site constituted of the glutamic acid and the two histidines at amino acids 341, 335 and 371 [10], respectively, appeared unaltered but are likely to be encapsulated within the mutant tertiary structure, thus failing to allow access of homogentisic acid to the catalytic site. Overall, deletion of exon 2 from the HGD protein predicts a significant conformational change in protein structure and a resulting severe AKU phenotype caused by abnormal HGD enzymatic activity.

It is worthwhile noting the absence of repetitive DNA elements flanking the deletion breakpoints. While it is known that recombination occurs between homologous sequences flanking a duplicated/deleted genomic segment [11], it does not appear to be the case for this 649 bp deletion. Thus, a clear mechanism for its emergence remains elusive. The report of this novel mutation in the HGD gene suggests that it could have either recently arisen and did not spread throughout the region or that it may be common but mutation screening of the HGD gene in AKU has not yet been extensively carried out in this area. The identification of novel AKU mutations in Jordan [12] along with this novel 649 bp deletion suggests that AKU in the Middle East may have independent origins than in other parts of the world. Thus, screening for the 649 bp deletion and other mutations in nearby populations would shed further light on the emergence of HGD in this part of the world. An important consideration in this endeavor would be the analysis of haplotypes on which the 649 bp deletion lies. The haplotype(s) on which this mutation resides may provide a valuable starting point to extend haplotype analysis of AKU in this region. The genetic admixture of ethnic groups in Lebanon, most of whom originated from distant regions in the Middle East and North Africa, could facilitate this endeavor and shed light on the origins of AKU that dates back to 1500 B.C. as reported by the discovery of homogentisic acid in an ochronotic Egyptian mummy [13]. In turn, these haplotypes will allow their integration into databases that will contribute to retracing ancient population migrations in the area.
